# Neural changes related to motion processing in healthy aging

**DOI:** 10.1016/j.neurobiolaging.2017.05.018

**Published:** 2017-09

**Authors:** Stefanie C. Biehl, Melanie Andersen, Gordon D. Waiter, Karin S. Pilz

**Affiliations:** a School of Psychology, University of Aberdeen, Aberdeen, UK; b Aberdeen Biomedical Imaging Centre, The Institute of Medical Sciences, University of Aberdeen, Aberdeen, UK

**Keywords:** Healthy aging, fMRI, Motion processing, Radial motion, Biological motion

## Abstract

Behavioral studies have found a striking decline in the processing of low-level motion in healthy aging whereas the processing of more relevant and familiar biological motion is relatively preserved. This functional magnetic resonance imaging (fMRI) study investigated the neural correlates of low-level radial motion processing and biological motion processing in 19 healthy older adults (age range 62–78 years) and in 19 younger adults (age range 20–30 years). Brain regions related to both types of motion stimuli were evaluated and the magnitude and time courses of activation in those regions of interest were calculated. Whole-brain comparisons showed increased temporal and frontal activation in the older group for low-level motion but no differences for biological motion. Time-course analyses in regions of interest known to be involved in both types of motion processing likewise did not reveal any age differences for biological motion. Our results show that low-level motion processing in healthy aging requires the recruitment of additional resources, whereas areas related to the processing of biological motion processing seem to be relatively preserved.

## 1 Introduction

Research in healthy aging has increasingly extended its scope from investigating higher cognitive functions to all areas of perception, and it has been shown that a variety of cognitive and perceptual abilities are affected by healthy aging (Grady, 2012, Greenwood, 2007, Hedden and Gabrieli, 2004). It has been proposed that an attenuation in neuromodulation and an increase in neural noise with increasing age are possible causes of the observable decline in abilities (Li et al., 2001).

Motion perception is an important and vital visual ability, which helps us to safely navigate through the environment. Previous studies have shown that low-level motion perception declines with healthy aging (Hutchinson et al., 2012). In an early study, Buckingham et al. (1987) found a deterioration of movement sensitivity with increasing age. In addition, motion detection and direction discrimination (Bennett et al., 2007) as well as speed perception (Atchley and Andersen, 1998, Norman et al., 2003, Norman et al., 2010) have been shown to be impaired. Older participants furthermore exhibit reduced spatiotemporal integration for apparent motion, requiring both shorter interstimulus intervals and smaller spatial displacements to reach accuracy comparable to younger adults (Roudaia et al., 2010). Moreover, motion direction discrimination thresholds have been found to continually increase with increasing age (Billino et al., 2008, Velarde et al., 2012). These age-related changes in low-level motion perception have been related to loss in sensitivity, increased spontaneous noise, and increased excitability of neurons in early visual areas, potentially related to a decrease in the inhibitory neurotransmitter gamma*-*aminobutyric acid (Leventhal et al., 2003, Schmolesky et al., 2000).

In addition to decreased low-level motion perception, more recent studies have also shown age-related changes in biological motion perception tasks. Biological motion perception is commonly investigated using point-light walkers (Johansson, 1973). Point-light walkers are stimuli that consist of local dots that represent the joints of a moving person. Each dot has a local motion trajectory that represents the movement of its corresponding joint over time. By integrating the local motion signals of all dots, the biological motion of the figure becomes apparent (for a review see Blake and Shiffrar, 2007). Previous research has suggested that biological motion is processed in 2 interacting neural pathways, the dorsal and the ventral pathway. The dorsal pathway is thought to process biological motion primarily based on the local motion information of the single dots, integrating information from local motion detectors in V1/V2 and middle temporal area hMT+ into optic flow detectors, further into optic flow pattern neurons in the superior temporal sulcus (STS). The ventral pathway is thought to process biological motion primarily based on the global form information, which is achieved by integrating the local dots into a global figure at any given point in time. Information from both pathways is thought to be integrated in motion pattern neurons in areas such as STS that allow for the decoding of the underlying movement (Giese and Poggio, 2003, Lappe, 2012).

In line with the previously mentioned model, STS activation has been shown to be specific to the perception of biological motion, while hMT+ has been shown to respond to visual motion more generally (Grossman et al., 2000, Morrone et al., 2000, Schultz and Pilz, 2009). Furthermore, areas previously related to static face processing—the occipital face area (OFA) and the fusiform face area (FFA)—have been found to show increased activation to point-light walkers (Grossman and Blake, 2002), but also to moving faces (Schultz and Pilz, 2009, Schultz et al., 2013). Michels et al. (2005) investigated brain structures responding preferentially to normal biological motion point-light walkers compared to point-light walkers devoid of local motion trajectories. Although STS showed a preferential response for the normal point-light walkers, it was still active during the presentation of the point-light walkers that contained primarily global form information. The same was true for FFA and OFA. These 3 areas consequently seem to be specifically attuned to the perception of biological motion as well as biological form information. Psychophysiological studies furthermore suggest that input from only the form pathway is sufficient to perceive biological motion, as participants still reached high discrimination accuracy for point-light walkers with no local motion information (Beintema and Lappe, 2002, Beintema et al., 2006). To summarize, biological motion can be processed using both the global motion and the form that is contained in the stimulus, and it has been suggested that both the ventral and the dorsal pathway are involved in the processing of biological motion. Information from both pathways is thought to be integrated in STS.

As for the biological motion perception, previous studies have also shown decreased biological motion perception for point-light walkers masked in a cloud of noise dots for older compared to younger adults (Billino et al., 2008, Pilz et al., 2010). However, it seems that older adults' performance for biological motion tasks improves with increased stimulus duration (Norman et al., 2004, Pilz et al., 2010, Spencer et al., 2016), suggesting that changes in biological motion perception might in part be related to increased processing times. In addition, older adults seem to use different strategies when processing biological motion, as performance for less familiar stimuli such as inverted point-light walkers improves for stimuli that primarily contain form information compared to those that contain both local motion and global form information: Pilz et al. (2010) asked older and younger participants to discriminate the walking direction of point-light walkers that contained primarily local motion, primarily global form, or both local motion and global form information. For upright walkers, older adults performed similarly to younger adults, especially at longer stimulus durations. When the walkers were inverted, older adults' performance for walkers containing both local motion and global form information was worse than performance of younger adults even for stimulus durations extending 3 seconds. However, older adults performed as well as younger adults for inverted walkers that primarily contained the global form information. These results suggest that brain areas primarily involved in motion processing are more affected by aging than areas primarily involved in form processing.

Age-related decline in visual processing has been hypothesized to be caused by neural changes leading to increased neural noise within the visual system (Betts et al., 2007). This is supported by animal studies finding decreased selectivity of V1 cells in senescent monkeys, which is possibly caused by reduced intracortical inhibition (Leventhal et al., 2003, Schmolesky et al., 2000). However, so far, the neural mechanisms underlying age-related changes in visual motion processing are relatively unknown. Therefore, this study investigated age-related changes in the neural activation of areas related to the perception of both low-level and biological motion. Changes in brain areas commonly related to low-level motion processing (hMT+) were investigated by showing participants a circular display of dots with alternating radial outwards and inwards motion, and a circular display of static dots. Neural activation related to biological motion processing (brain areas STS, FFA, OFA) was investigated by showing participants displays of normal (Johansson, 1973), random position (Beintema and Lappe, 2002, Beintema et al., 2006), and scrambled point-light walkers (Pilz et al., 2010, Vaina et al., 2001) performing different actions (Vanrie and Verfaillie, 2004).

Based on the previous neuroimaging and behavioral results regarding low-level and biological motion perception, we expected to find less efficient neural activation especially in the dorsal area hMT+ for both low-level and biological motion for older compared to younger participants. The processing of low-level motion stimuli heavily relies on processing in this area, whereas the processing of biological motion stimuli is thought to involve both dorsal area hMT+ and ventral areas FFA and OFA related to motion and form information, respectively, as well as STS (Giese and Poggio, 2003). Given the previously mentioned age-related behavioral changes in low-level motion perception, and altered processing of biological motion stimuli containing local motion information, it is likely that dorsal area hMT+ is especially prone to age-related decline.

## 2 Methods

### 2.1 Participants

All participants attended a screening appointment to assess their near and far vision, and obtain handedness data (Oldfield, 1971) as well as sociodemographic information, visual health, and relevant medical history. Only right-handed participants with near and far vision above 16/20, no history of cataract, glaucoma, or maculopathy, and an eye exam within the last 3 years were included in the study. To ensure that none of the older participants were suffering from mild cognitive impairment, the older group additionally completed the Montréal Cognitive Assessment (Nasreddine et al., 2005). As depression in old age was previously linked to executive (and visuospatial) impairment (Butters et al., 2004), older participants were screened for depressive symptomatology using the short version of the Geriatric Depression Scale (Sheikh and Yesavage, 1986). The cutoff scores were ≥26 for the MoCa and ≤10 for the Geriatric Depression Scale, as recommended by the respective authors.

A total of 21 younger participants (15 women, age range 20–30 years) and 20 older participants (12 women, age range 62–78 years) took part in the functional magnetic resonance imaging (fMRI) experiment. From this sample, 1 older participant had to be excluded because his structural scan showed an arachnoid cyst in the lower occipital cortex, and 2 younger participants had to be excluded because substantial movement after the initial slice alignment placed the lower portion of the occipital lobes outside the field of view and a technical error prevented the recording of responses to the attention task, respectively. The final sample thus consisted of 19 older (mean age 68.8 years, SD 4.5) and 19 younger (mean age 23.3 years, SD 3.0) participants. Ethical approval was obtained through the School of Psychology Ethics Committee of the University of Aberdeen, and the study was registered with the National Health Service Grampian Research and Development Office (NHS Grampian R & D; Project Number, 2014PC006); all procedures involved were in accordance with the Declaration of Helsinki (World Medical Association, 2001). Participants gave written informed consent after having received a full explanation of the procedures.

### 2.2 Experimental paradigms

The low-level motion paradigm comprised 3 conditions, presented in blocks of 10 seconds with interblock intervals of 2 seconds: a circular display of dots showing alternating radial outwards and inwards motion, a circular display of static dots, and fixation. In the moving dots condition, 500 dots with a diameter of 0.2° visual angle moved on an inwards or outwards trajectory at a speed of 3 degrees/second inside a circle with a radius of 15° visual angle. Motion trajectories were reversed every second. Presentation rate was 25 frames/second and all dots had a limited lifetime in that 3% of dots were extinguished and replaced at a random position within the circle at every frame.

**Fig. 1:**
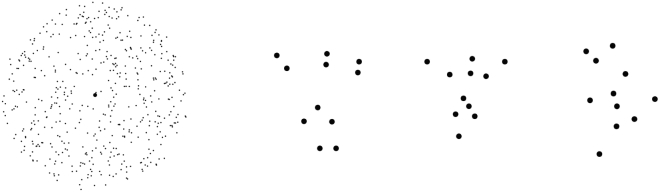
Experimental stimuli. Exemplary stimuli of the low-level motion paradigm (left) and biological motion paradigm (right), showing (from left to right) a normal, a random position, and a scrambled front view of a point-light walker playing tennis. Stimulus color is reversed for better visibility.

The biological motion paradigm consisted of 3 different conditions: normal, random position, and scrambled point-light walkers performing 1 of 13 different actions (these actions were: crawling, cycling, drinking, driving, mowing, painting, pedaling, playing pool, saluting, sawing, using a spade, stirring, and tapping) (Vanrie and Verfaillie, 2004). In the “normal” condition, point-light walkers contained both the local motion of the single dots and the global form of the figure. In the “random position” condition, the local motion information of the stimulus was impaired while the form information remained intact. In contrast, in the “scrambled” condition, the individual motion trajectories remained intact while the form information of the stimulus was decreased (see Fig. 1 for exemplary low-level motion and biological motion stimuli). Stimuli were presented centrally, with random displacements of 100 pixels to the left and right/top and bottom of the fixation dot. Block duration was 16 seconds, followed by 6 seconds of fixation. Eight randomly selected stimuli were presented per block with a presentation duration of 1000 ms and an interstimulus interval of 1000 ms.

The functional localizer consisted of a moving faces paradigm (Schultz and Pilz, 2009) (object images: courtesy of Michael J. Tarr, Center for the Neural Basis of Cognition and Department of Psychology, Carnegie Mellon University, http://www.tarrlab.org/; face stimuli: Amsterdam Dynamic Facial Expression Set (van der Schalk et al., 2011)) with 5 different conditions: moving faces, static faces, phase-scrambled faces, objects, and fixation. Block duration was 16 seconds, with a 2 seconds interblock interval. Eight stimuli were presented per block with a presentation duration of 1000 ms and an interstimulus interval of 1000 ms. All videos contained 25 frames, started with a neutral expression and ended at the peak of the expression. Static faces consisted of the last frame of this sequence. The background of all images and videos consisted of white noise.

To equalize attention across the different conditions, participants were instructed to focus on a centrally presented fixation dot and to indicate an irregularly occurring white-to-red color change via button press in all paradigms.

### 2.3 Image acquisition and analysis

All images were acquired on a 3T Philips Achieva X-Series MRI scanner, using a 32-channel phased-array head coil. For all paradigms, the repetition time of the T2*-weighted gradient echo planar imaging (EPI) sequence was 1.9 seconds, the echo time was 30 ms, and the flip angle was 70°. Thirty-five axial slices aligned to the anterior commissure-posterior commissure line were acquired in sequential descending order (0.5 mm gap), voxel size was 3 × 3 × 2.5 mm^3^. A high-resolution structural T1 scan was acquired for each participant (160 slices; voxel size 1 × 1 × 1 mm^3^). All experiments were programmed using the MATLAB (The MathWorks, Inc, Natick, MA, USA) based Psychtoolbox extension (Brainard, 1997, Kleiner et al., 2007, Pelli, 1997). Participants viewed the stimuli on a back-projection screen via a mirror attached to the head coil.

All fMRI data were analyzed using statistical parametric mapping (SPM8; Wellcome Trust Centre for Neuroimaging, London, UK) implemented in MATLAB: EPI images were realigned and image acquisition time was corrected to the middle slice. The structural scan was coregistered to the mean EPI image and segmented. EPI images were normalized with 2 mm^3^ voxel size and smoothed with an 8 mm^3^ full-width at half maximum Gaussian smoothing kernel. Subsequently, we performed first level analyses incorporating all conditions of interest as well as the 6 movement parameters obtained during preprocessing. To investigate whole-brain differences between the younger and the older group, second-level analyses were carried out using 2-sample *t*-tests. Data were thresholded at *p*_FWE_ <0.05 at cluster level, with *p* < 0.001 as cluster-forming threshold.

We identified motion sensitive regions of interest (ROI) in hMT+ using the contrast moving > static dots and in higher-level regions using the contrast moving > scrambled faces. For the ROI analysis, ROIs were defined as functional masks resulting from activated clusters from each participant's individual general linear model (GLM) analysis—areas in occipital gyrus and fusiform gyrus corresponding to classic face sensitive regions OFA and FFA, and areas in posterior STS corresponding to regions responsive to biological motion (Grossman et al., 2000, Schultz and Pilz, 2009, Schultz et al., 2013). In order to standardize the process of ROI definition across all participants, a fixed statistical threshold was chosen based on the overall strength of the activation for the given paradigm. Single-participant GLMs were thus thresholded at *p* < 0.001 for the functional localizer and *p*_FWE_ <0.05 for hMT+. For each participant, the area of interest was defined using a spherical mask with a diameter of 6 mm that was centered on the peak voxel of the individually defined cluster. Block-averaged response time courses of each condition in each ROI were computed by extracting raw BOLD signal data that was filtered to remove low frequencies (cutoff 128 seconds), then averaged over the voxels in each ROI. The block-related responses to each condition were converted into percent signal change from average activity and averaged for each participant from 10 seconds before to 30 seconds after each block onset. For each condition, data was baseline corrected by averaging the activation from 5.3 to 0.5 seconds before stimulus onset and subtracting it from each subsequent time point.

## 3 Results

### 3.1 Behavioral results

Participants correctly detected around 70% of color changes with false alarms showing some variation between paradigms, but no clear clustering in any one condition except in the “moving faces” condition of the functional localizer. As an index of target detection performance, d' was calculated (Bendixen and Andersen, 2013) and participants performed above chance with d' >1 in all paradigms (see Table 1).

**Table 1:** Means and standard deviations (SD) of d' as well as false alarms (FA) for the attention task for all paradigms and age groups

Age group	Low-level motion, mean (SD)		Biological motion, mean (SD)		Functional localizer, mean (SD)	
	d'	FA	d'	FA	d'	FA
Younger adults	2.77 (0.32)	1.4 (1.5)	2.15 (0.13)	10.7 (2.8)	2.32 (0.21)	8.7 (3.2)
Older adults	2.62 (0.39)	2.4 (2.5)	2.19 (0.31)	9.4 (5.2)	2.11 (0.32)	12.5 (12.3)

Two mixed model analyses of variance (ANOVAs) with the between-subjects factor “age group” (younger vs. older participants) and the within-subjects factor “task” (low-level motion paradigm, biological motion paradigm, functional localizer) were conducted for d' and number of false alarms to examine behavioral performance differences across age groups and task conditions. The ANOVA for d' showed a main effect of condition (F_(2,70)_ = 58.37, *p* < 0.001), with significantly higher d' during the low-level motion paradigm than during the biological motion paradigm (t_(36)_ = 8.80, *p* < 0.001) and during the functional localizer (t_(36)_ = 8.89, *p* < 0.001), which were not significantly different (*p* = 0.47). There was no main effect of age group (*p* = 0.19) and no interaction (*p* = 0.09). The ANOVA for number of false alarms also showed a main effect of condition (F_(2,70)_ = 34.38, *p* < 0.001), with significantly more false alarms during the biological motion paradigm and during the functional localizer than during the low-level motion paradigm (t_(36)_ = 13.90, *p* < 0.001 and t_(36)_ = 6.29, *p* < 0.001, respectively). Number of false alarms for the biological motion paradigm and the functional localizer were not significantly different (*p* = 0.75). There was no main effect of age group (*p* = 0.40) and no interaction (*p* = 0.12).

### 3.2 fMRI results

#### 3.2.1 Low-level motion paradigm

##### 3.2.1.1 Whole-brain analysis

**Fig. 2:**
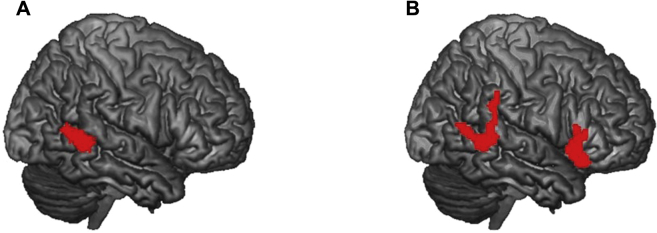
Between-group comparisons. Clusters of significantly increased activation in the older group during the static (A) and the moving (B) condition. Data were thresholded at *p*_FWE_ <0.05 at cluster level.

Within-group second-level analyses showed robust activation in lateral occipital and hMT+ areas for moving compared to static stimuli in both age groups (see Table 2). Between-group comparisons of the activation furthermore indicated 3 clusters showing significant age differences (see Fig. 2 and Table 2). For the static stimuli, the older group showed significantly higher activation in an area of the right middle temporal gyrus (cluster size 343 voxels) than the younger group. For the moving stimuli, the older group also showed significantly higher activation in the right middle temporal gyrus (cluster size 398 voxels), and the right inferior frontal gyrus (cluster size 414) than the younger group.

**Table 2:** Significant clusters and trends in within-group and between-group comparison for given paradigm, contrast of interest, and age group for the whole-brain analyses

Paradigm	Contrast	Age group	Anatomical region	*p*_FWE_ cluster level	MNI coordinates
Low-level	Moving > static	Young	Middle occipital gyrus R^a^	<0.001	38, −86, 4
			Middle temporal gyrus R^a^	<0.001	44, −62, 0
			Middle occipital gyrus R^a^	<0.001	24, −88, 2
		Older	Inferior occipital gyrus L	<0.001	−32, −88, −6
			Middle occipital gyrus L	<0.001	−12, −100, 2
			Inferior occipital gyrus L	<0.001	−42, −82, −8
			Middle occipital gyrus R^a^	<0.001	24, −92, 8
			Middle occipital gyrus R^a^	<0.001	32, −88, 8
			Middle temporal gyrus R^a^	<0.001	50, −66, 4
	Static	Older > young	Middle temporal gyrus R	0.02	62, −44, 4
	Moving	Older > young	Middle temporal gyrus R	0.004	56, −56, 10
			Inferior frontal gyrus R	0.004	50, 22, 4
Biological	Normal > scrambled	Young	No significant clusters		
		Older	No significant clusters		
	Normal > random	Young	Middle occipital gyrus L	0.02	−32, −96, 4
			Inferior occipital gyrus L	0.07	−40, −74, −6
		Older	Middle occipital gyrus L	0.001	−34, −84, 8

a Marks the clusters used in the additional time-course analyses for the biological motion paradigm.

##### 3.2.1.2 Time-course analysis

hMT+ could be identified bilaterally in all participants. Mean activation across both hemispheres was used for a mixed model ANOVA with the between-subjects factor “age group” (younger vs. older participants) and the within-subjects factor “condition” (moving, static). The ANOVA showed a main effect of condition (*F*_(1,36)_ = 131.21, *p* < 0.001) with significantly more activation during the moving than during the static condition. There was no main effect of age group (*p* = 0.27) and no interaction (*p* = 0.50).

#### 3.2.2 Biological motion paradigm

##### 3.2.2.1 Whole-brain analysis

Within-group second-level analyses for “normal” versus “scrambled” stimuli showed no activation above the significance threshold in any of the 2 groups. Between-group comparisons of the activation furthermore indicated no clusters showing significant age differences.

In contrast, within-group second-level analyses for the “normal” versus the “random” stimuli showed significantly increased activation in the left middle occipital gyrus in both groups (see Table 2). However, between-group comparisons indicated no clusters showing significant age differences.

##### 3.2.2.2 Time course analysis

###### 3.2.2.2.1 hMT+

**Fig. 3:**
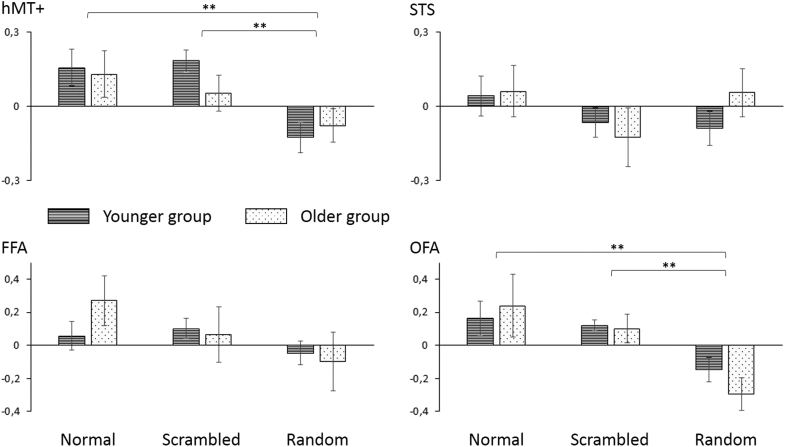
Time-course analysis. Mean percent signal change in the different conditions of the biological motion paradigm for the 2 age groups. hMT+, both hemispheres (mean); STS, right hemisphere; FFA, right hemisphere; OFA, left hemisphere. Error bars denote standard error of the mean. Significant differences are marked with ** for *p* < 0.01. Abbreviations: FFA, fusiform face area; OFA, occipital face area; STS, superior temporal sulcus.

This ROI could be identified bilaterally in all participants. Mean activation across both hemispheres was used for a mixed model ANOVA with the between-subjects factor “age group” (younger vs. older participants) and the within-subjects factor “condition” (normal, scrambled, random). The ANOVA showed a main effect of condition (*F*_(2,70)_ = 5.74, *p* = 0.005), with significantly higher activation during the normal compared to the random condition (*t*_(36)_ = 2.94, *p* = 0.006) as well as during the scrambled compared to the random condition (*t*_(36)_ = 3.03, *p* = 0.005). There was no main effect of age group (*p* = 0.33) and no interaction (*p* = 0.53; see Fig. 3).

Additional time-course analyses were carried out on the 3 clusters showing increased activation for the contrast moving versus static stimuli in the within-group comparison in both groups (see Table 2). While 2 of the 3 ROIs from the within-group comparison showed an increased response to moving stimuli (i.e., normal and scrambled point-light walkers), neither the clusters located in the right middle occipital gyrus nor the cluster located in the middle temporal gyrus showed a main effect of group or an interaction of group and condition.

###### 3.2.2.2.2 Superior temporal sulcus

Similar to previous research (Michels et al., 2005), this ROI could only be identified reliably in the right hemisphere of 11/19 younger and 11/18 older participants. The statistical analysis showed no main effect of age group (*p* = 0.37), no main effect of condition (*p* = 0.38), and no interaction of these 2 factors (*p* = 0.62).

###### 3.2.2.2.3 Fusiform face area

This ROI could be identified in the right hemisphere of 14/17 younger and 12/18 older participants and in the left hemisphere of 13/17 younger and 10/18 older participants. In order to analyze a larger sample, analyses were restricted to the right hemisphere. The statistical analysis showed no main effect of age group (*p* = 0.45), no main effect of condition (*p* = 0.25), and no interaction of these 2 factors (*p* = 0.58).

###### 3.2.2.2.4 Occipital face area

This ROI could be identified in the right hemisphere of 13/17 younger and 11/18 older participants and in the left hemisphere of 14/17 younger and 12/18 older participants. In order to analyze a larger sample, analyses were restricted to the left hemisphere. The statistical analysis showed a main effect of condition (*F*_(2,48)_ = 6.78, *p* = 0.005), with significantly higher activation during the normal compared to the random condition (*t*_(25)_ = 2.90, *p* = 0.008) as well as during the scrambled compared to the random condition (*t*_(25)_ = 3.65, *p* = 0.001). There was no main effect of age group (*p* = 0.56), and no interaction of these 2 factors (*p* = 0.66).

Correlating d' with peak ROI activations during the low-level and the biological motion tasks yielded no significant results.

## 4 Discussion

This is to our knowledge the first fMRI study investigating the neural correlates of motion perception in healthy human aging. The effect of aging on low-level motion processing and biological motion processing was investigated in a group of younger and older adults with a mean age difference of 45.5 years.

We found significant age-related differences in the processing of simple radial motion. Although peak activation was similar for both age groups, the second level between-group analysis showed that older participants recruited a more extensive area of middle/superior temporal gyrus for radial motion. In addition, older participants also showed activation in an area of right inferior frontal gyrus, which was inactive in younger adults. Increased activation in this area was found before in a sample of younger adults viewing biological motion stimuli with reduced motion information (Michels et al., 2005). This was interpreted as possibly reflecting increased effort caused by the more complex nature of the unusual motion stimuli, and a similar explanation might be appropriate for our finding. As this is a cross-sectional study, it is possible that the younger group's higher familiarity with computer generated low-level motion (e.g., in computer games) allowed them to focus on the attentional task more readily, thus expending less overall effort than the older group. However, studies on face processing also report increased frontal activation in older compared to younger adults (Goh et al., 2010, Gunning-Dixon et al., 2003, Lee et al., 2011). While Gunning-Dixon et al. (2003) interpreted this increased frontal activation as possibly reflecting increased task-related effort, Lee et al. (2011) used an adaptive behavioral task and found a positive association of frontal recruitment and better performance in the older group. Furthermore, Goh et al. (2010) reported overall increased frontal activation in an older compared to a younger group during a face adaptation task in the absence of behavioral performance differences. In line with these findings, older adults' increased frontal activation in our study might reflect compensatory activation for an age-related decline in overall cognitive functioning—specifically affecting low-level motion processing—as detailed in the scaffolding theory (Lee et al., 2011, Park and Reuter-Lorenz, 2009). Future studies of motion processing might therefore benefit from employing additional analysis methods such as dynamic causal modeling to identify functional links during task completion.

Even though we found age-related differences in hMT+ activation for low-level motion, this area did not show age-related differences for biological motion as tested with scrambled, normal, and random-position point-light walkers. For both age groups, we replicated previous imaging results of increased sensitivity of hMT+ to low-level motion independent of the global stimulus form Grossman et al. (2000). In addition, areas traditionally associated with static face processing (Rossion et al., 2003) showed increased activation for biological motion in both age groups. The OFA showed increased activation to normal and scrambled, but not to random point-light walkers. Findings for FFA and for STS are less clear. Results nominally indicate larger activation for normal (FFA) and for normal and random (STS) stimuli than for scrambled biological motion. However, these differences did not reach statistical significance, which might be due to the reduced sample size in the ROI analyses. Thus, future studies should investigate larger samples to obtain higher power for these difficult to define higher-level regions of interest.

While STS can be difficult to locate in some participants (Michels et al., 2005), detection of FFA and OFA was more arduous in the older group. This is in line with a previous study on face processing in healthy aging which reports a dedifferentiation in the activation of face-specific regions (Burianova et al., 2013), presumably making it more difficult to functionally locate these areas with the predefined contrasts. Future studies might thus consider employing different thresholds or different contrasts for the functional ROI definition in younger and older participants, although this would come at the high cost of reduced consistency in the analyses across groups. In addition, it might be worthwhile to include a localizer paradigm for body selective regions in future studies. While previous studies reported increased sensitivity of FFA/OFA to biological motion (Grossman and Blake, 2002, Michels et al., 2005) there is also some indication of increased extrastriate body area sensitivity (Michels et al., 2005), as well as of the FFA sensitivity being driven by an overlap of FFA and the fusiform body area (Peelen et al., 2006). Alternatively, future studies could strive to develop a localizer contrasting point-light walkers with face and/or body stimuli to identify areas which are particularly sensitive to the point-light stimuli.

Our findings are in line with the results of previous psychophysiological studies: Age-related decline has been shown to strongly affect tasks of low-level motion (Bennett et al., 2007, Billino et al., 2008, Buckingham et al., 1987, Hutchinson et al., 2012, Norman et al., 2003, Roudaia et al., 2010), which in turn are strongly related to activation in dorsal area hMT+. In contrast, the perception of biological motion has been found to remain relatively intact in healthy aging (Billino et al., 2008, Norman et al., 2004, Pilz et al., 2010, Spencer et al., 2016), possibly because there is an increasing reliance on form perception over motion processing with increasing age (Pilz et al., 2010).

It seems somewhat surprising that the results did not show increased STS activation for the normal compared to the scrambled condition as this has previously been reported in the literature (Grossman and Blake, 2002, Grossman et al., 2000, Grossman et al., 2010, Vaina et al., 2001). There are a few potential explanations for this: first and foremost, it has to be noted that the behavioral task in our study was stimulus-unrelated. This served to avoid potentially confounding effects of attentional allocation to the stimuli in the different conditions and also across age groups. Such attention-related differences would have confounded with any differences in neural activation between age groups or conditions. In line with the rationale guiding this design, no correlations between behavioral task performance and stimulus-related activation were found for any of the 2 groups. Thus, alternative interpretations related to distraction suppression and interference control can be excluded with reasonable confidence.

However, tasks in the behavioral studies mentioned above were mostly stimulus-related, and activation in STS for normal compared to scrambled conditions was mostly found in studies where attention was allocated to the stimulus. Grossman et al., for example, asked participants to perform a 1-back task that directed participants' attentional focus on the presented stimuli (Grossman and Blake, 2002), and Michels et al. (2005) asked participants to perform a stimulus-related forced choice discrimination task. Therefore, focusing attention away from the stimuli in our study might have attenuated potentially existing differences between these 2 stimulus categories. It is therefore possible that an increased sample size might have been required to obtain the same results as previous studies, which focused attention directly on the presented stimuli. Second, the addition of random point-light walkers besides the usually tested normal and scrambled point-light walkers might have influenced the results. The only other fMRI study that previously investigated random point-light walkers did not use scrambled walkers (Michels et al., 2005), and as the ROIs in this study were defined based on anatomical landmarks, these results are very difficult to compare to the results obtained here.

While previous studies on dynamic facial motion still found the hypothesized effects despite a very difficult task that was unrelated to the presented stimuli (Schultz et al., 2013), effects were more pronounced when the behavioral task increased attentional focus on the presented stimuli (Schultz and Pilz, 2009). Although future studies might benefit from focusing the behavioral task on the presented stimuli while still taking care to avoid attentional biases in the different conditions, finding such a task that furthermore causes no major performance differences between the two age groups might not be possible. It further has to be noted that the behavioral task employed here was a simple target detection task with participants showing hit rates of around 70%. In line with attentional load theory as well as corresponding previous research (Lavie and Tsal, 1994, Rees et al., 1997), it should therefore be assumed that participants were easily able to focus on the task at hand while also processing the simultaneously presented task-irrelevant motion stimuli. As previous research points to an age-related decline in the processing and tracking of multiple objects (Stormer et al., 2013), it might indeed be preferable to present a very simple central task while investigating stimuli presented “in the background”. However, as psychophysiological studies of biological motion perception point to a possibly age-related decrease in direction discrimination with increasing stimulus complexity (Pilz et al., 2010), it might also be promising to employ stimulus material of increased complexity (e.g., inverted point-light walkers or point-light walkers in random noise) to test if neural processing under these conditions still remains unchanged with increasing age.

## 5 Conclusion

This first fMRI study of motion perception in healthy aging supports the notion of an age-related change in low-level motion processing. Our results indicate that this change in visual area hMT+ necessitates the recruitment of additional regions in the temporal and frontal cortex for processing low-level motion. In contrast, there is little activation difference between younger and older adults in areas related to biological motion processing. This supports the hypothesis that the processing of biological motion is less affected by healthy aging than the processing of low-level motion.

## Disclosure statement

The authors have no actual or potential conflicts of interest.
